# Supplementary material for: The urban built environment and adult BMI, obesity, and diabetes in Latin American cities
Source: Nat Commun. 2022 Dec 29;13:7977. doi: 10.1038/s41467-022-35648-w (PMC9800402; doi:10.1038/s41467-022-35648-w)
Supplement: Supplementary file 1 — Supplementary Information [file 41467_2022_35648_MOESM1_ESM.pdf]

Supplementary Materials for

The urban built environment and adult BMI, obesity and diabetes in Latin American cities

Cecilia Anza-Ramirez*, Mariana Lazo, Jessica Hanae Zafra-Tanaka, Ione Avila-Palencia, Usama Bilal, Akram Hernández, Carolyn Knoll, Nancy Lopez-Olmedo, Mónica Mazariegos, Kari Moore, Daniel A. Rodriguez, Olga L. Sarmiento, Dalia Stern, Natalia Tumas, J. Jaime Miranda*
*Correspondence to: cecilia.anza@upch.pe, jaime.miranda@upch.pe

Table of Contents

Supplementary Table 1 | Sample size by country for BMI and obesity and Type 2 Diabetes 1

Supplementary Table 2 | Characteristics of the sample by country..2

Supplementary Table 3 | Characteristics of the sample by population density quartiles 4

Supplementary Table 4a | Sensitivity analysis 5

Supplementary Table 4b | Sensitivity analysis 6

Supplementary Table 4c | Sensitivity analysis 7

Supplementary Table 5 | General information of the health surveys used for individual level data ...8

Supplementary Table 6 | Description of exposure and covariates variables used............................11

Supplementary Table 7 | Correlation matrix among variables of interest...14

Supplementary Table 1 | Sample size by country for BMI and obesity and Type 2 Diabetes
Sample size for each country is displayed as observations are lost due to incomplete or missing data.

**Outcomes**		**BMI and Obesity**					**Type 2 Diabetes**				
**Country**	**Survey sample size**	**Individuals with complete sub-city unit data**	**Individuals selected for anthropometry module**	**Individuals with complete outcome data**	**Individuals with complete built environment data**	**Individuals with complete socio-demographic data (final sample size)**	**Individuals with complete sub-city unit data**	**Individuals selected for health module**	**Individuals with complete outcome data**	**Individuals with complete built environment data**	**Individuals with complete socio-demographic data (final sample size)**
Argentina	21,573	21,451	21,451	20,184	20,184	**20,184**	21,451	**21,451**	21,337	21,337	**21,337**
Brazil	93,113	67,321	29,353	25,528	25,528	**25,528**	67,321	**67,353**	24,966	24,966	**24,966**
Chile	3,146	3,140	3,140	2,866	2,866	**2,861**	3,140	**3,140**	3,107	3,107	**3,106**
Colombia	49,521	49,521	5,934	5,886	5,886	**5,757**	49,521	**18,655**	18,653	18,653	**18,653**
Costa Rica	1,427	0	0	0	0	**0**	0	**0**	0	0	**0**
Guatemala	1,397	1,397	1,397	1,040	1,040	**1,040**	1397	**1,397**	1382	1382	**1382**
Mexico	72,867	72,789	25,171	22,895	22,895	**22,895**	72,789	**26,335**	26,335	26,335	**26,335**
Nicaragua	1,993	1,993	1,993	1,693	1,693	**1,692**	1,993	**1,993**	1,949	1,949	**1,948**
Panama	11,435	11,394	0	0	0	**0**	11,394	**11,394**	11,374	11,374	**11,063**
Peru	11,929	11,929	11,929	11,831	11,823	**11,821**	11,929	**11,929**	11,925	11,917	**11,915**
El Salvador	1,546	1,546	1,538	1,538	1,538	**1,502**	1546	**1,546**	1542	1542	**1506**
**Total**	**269,947**	**242,481**	**101,914**	**93,461**	**93,453**	**93,280**	**242,481**	**127,193**	**122,570**	**122,562**	**122,211**

Supplementary Table 2 | Characteristics of the sample by country
All variables of interest are shown by country.

Variables<br>Individual level variables	Argentina (21,337)	Brazil (24,966)	Chile (3,106)	Colombia (18,653)	Guatemala (1,382)	México (26,335)	Nicaragua (1,948)	Panamá (11,063)	Peru (11,915)	El Salvador (15,06)
**Age**	44.7 ± 18	43.9 ± 16.6	47 ± 17.7	39.3 ± 14.1	39.7 ± 15.5	43.6 ± 16.1	40.6 ± 14.9	41.4 ± 16.6	39.7 ± 15.7	45.7 ± 17
**Female sex**	56.2%	50.6%	59.4%	57.4%	67.2%	57.3%	49.2%	63.3%	57.5%	66.8%
**Highest educational level completed**										
Less than primary	9.3%	21.1%	11.3%	17.3%	37.1%	20.5%	6.2%	8.5%	12.8%	33.0%
Primary	36.9%	21.1%	34.2%	34.7%	45.0%	48.8%	33.5%	36.2%	17.4%	39.4%
Secondary	37.0%	38.8%	45.9%	39.1%	16.5%	21.0%	43.7%	38.2%	57.5%	22.9%
University	16.9%	19.1%	8.6%	8.8%	1.5%	9.7%	16.1%	17.2%	12.3%	4.7%
**Sub-city level variables**										
**Intersection density** (n/km^2)	19.2 ± 28.4	38.1 ± 31	33.6 ± 53.8	19.8 ± 19.6	58.4	21.1 ± 31.8	51.6	35.2 ± 40.7	48 ± 55.2	38.9 ± 32.2
**Greenness** (median NDVI)	0.6 ± 0.2	0.7 ± 0.1	0.5 ± 0.3	0.8 ± 0.1	0.7	0.6 ± 0.2	0.8	0.7 ± 0.2	0.4 ± 0.3	0.8 ± 0.1
**Population Density in built up areas** (n/km2)	5,276 ± 3,038	8,312 ± 3,605	7,068 ± 2,845	1,3640 ± 4,893	13,154	5,772 ± 2,913	8,458	6,496 ± 4,722	11,282 ± 5,092	12,017 ± 5,025
**Population educational attainment (Z-score)**	-0.3 ± 1.0	1.2 ± 0.8	-0.5 ± 1.0	0.1 ± 0.8	-1.7	-0.2 ± 1.2	-0.3	1.9 ± 2.6	2.2 ± 1.5	-1.0 ± 1.3
**City level variables**										
**Fragmentation** [Patch density (n/100ha)]	0.3 ± 0.3	0.6 ± 0.4	0.3 ± 0.2	0.4 ± 0.2	0.8	0.4 ± 0.3	1.2 ± 0	0.5 ± 0.1	0.2 ± 0.1	1.1 ± 0.3
**Isolation** [Mean distance to the nearest urban patch within the geographic boundary(m)]	88.9 ± 32.6	71.2 ± 10.9	93.5 ± 37.3	96.1 ± 57.2	67.5	94.3 ± 42.2	64.6 ± 0	70.2 ± 4.3	76 ± 14.4	66.7 ± 3.6
**Percentage of urban area** (%)	6.3 ± 7.2	11.7 ± 9.4	8.6 ± 11.1	7.9 ± 6.6	13.5	8 ± 7.2	12.1 ± 0	6 ± 1.6	7.7 ± 7.6	12.1 ± 5.5

Supplementary Table 2 (Cont.) | Characteristics of the sample by country.

Outcomes	Argentina (21,337)	Brazil (24,966)	Chile (3,106)	Colombia (18,653)	Guatemala (1,382)	México (26,335)	Nicaragua (1,948)	Panamá (11,063)	Peru (11,915)	El Salvador (15,06)
**BMI** (Kg/m^2)*	26.6 ± 5.4	26.6 ± 5.1	27.9 ± 5.4	25.3 ± 6.9	26.8 ± 4.9	28.4 ± 5.8	27.9 ± 5.6	--	27.4 ± 4.7	27.8 ± 5.3
**Obesity** (BMI ≥ 30 kg/m^2) *	21.1%	21.6%	29.2%	14.9%	23.8%	34.1%	30.9%	--	26.0%	30.6%
**Diabetes** (yes)	8.7%	6.7%	8.1%	3.8%	5.6%	9.8%	5.8%	6.4%	3.7%	9.5%

NDVI: Normalized Difference Vegetation Index. BMI: Body mass index. * Calculated from the obesity data set. Values are show as percentage or Mean ± Standard Deviation

Supplementary Table 3 | Characteristics of the sample by population density quartiles

Variables	Population density by quartiles<br>Low<br>Q1 (n = 30,615)<br>[1,024.1 - 4,740.2]	Mid-low<br>Q2 (n = 30,944)<br>[4,772.6 - 6,735.8]	Mid-high<br>Q3 (n = 30,491)<br>[6,736.0 - 10,512.2]	High<br>Q4 (n = 30,111)<br>[10,517.3 - 39,498.7]
**Individual level variables**				
**Age**	43.3 ± 16.9	42.9 ± 16.6	42.2 ± 16.2	42 ± 16.1
**Sex**				
Female	57.6%	58.6%	58.8%	59.1%
Male	42.4%	41.4%	41.2%	41.0%
**Highest educational level completed**				
Less than primary	16.5%	15.2%	16.8%	16.5%
Primary	39.3%	37.1%	28.9%	30.3%
Secondary	31.9%	32.5%	39.7%	41.1%
University or higher	12.4%	15.2%	14.6%	12.1%
**Sub-city level variables**				
**Intersection density** (n/km^2)	6.3 ± 11.9	19.5 ± 23	36.2 ± 34.4	56.1 ± 42.9
**Greenness** (median NDVI)	0.6 ± 0.2	0.7 ± 0.2	0.7 ± 0.2	0.6 ± 0.2
**Population Density in built up areas** (n/km^2)	3,724 ± 649	5,618 ± 544	8,410 ± 1,185	15,376 ± 3,943
**Population educational attainment (Z-score)**	-0.1 ± 1.4	0.5 ± 1.6	0.9 ± 1.7	0.8 ± 1.4
**City level variables**				
**Fragmentation** [Patch density (n/100ha)]	0.3 ± 0.3	0.4 ± 0.2	0.5 ± 0.3	0.6 ± 0.3
**Isolation** [Mean distance to the nearest urban patch within the geographic boundary(m)]	98 ± 38.6	83.3 ± 31.5	77 ± 19.4	77 ± 44.2
**Percentage of urban area** (%)	4.3 ± 5.5	6.3 ± 4.8	8.0 ± 5.9	15.2 ± 9
**Outcomes**				
**BMI** (Kg/m^2)*	27.4 ± 5.5	27.3 ± 5.6	27.1 ± 5.5	26.8 ± 5.5
**Obesity** (BMI ≥ 30 kg/m^2)*	26.6%	26.4%	24.1%	22.9%
**Diabetes** (yes)	8.3%	7.6%	6.3%	5.7%

NDVI: Normalized Difference Vegetation Index. BMI: Body mass index. * Calculated from the obesity data set. Values are show as percentage or Mean ± Standard Deviation

Supplementary Table 4a | Sensitivity analysis
Associations between sub-city and city exposures and BMI. Different sensitivity analyses are presented.

Sub-city and city characteristics (z-scores)	Full Model for BMI	Excluding Argentina	Excluding pop.dens >3SD	Recent surveys only	Excluding BMI outliers	Including interaction terms between country and individual educational level
	**Coeff [95% CI]**	**Coeff [95% CI]**	**Coeff [95% CI]**	**Coeff [95% CI]**	**Coeff [95% CI]**	**Coeff [95% CI]**
**Sub-city intersection density (**n/km2)	0.10 [0.02 ; 0.19]	0.09 [0.02 ; 0.16]	0.08 [-0.00 ; 0.17]	0.09 [-0.00 ; 0.18]	0.09 [0.01 ; 0.18]	0.09 [0.02 ; 0.17]
**Sub-city greenness** (median NDVI)	-0.04 [-0.15 ; 0.06]	-0.07 [-0.17 ; 0.04]	-0.05 [-0.15 ; 0.05]	-0.03 [-0.14 ; 0.07]	-0.04 [-0.15 ; 0.07]	-0.05 [-0.14 ; 0.05]
**Sub-city population density in built up areas (**n/km2)	-0.03 [-0.14 ; 0.08]	0.02 [-0.08 ; 0.12]	-0.01 [-0.11 ; 0.09]	0.00 [-0.12 ; 0.12]	-0.03 [-0.14 ; 0.09]	-0.01 [-0.11 ; 0.08]
**City fragmentation** [Patch density (n/100ha)]♯	-0.13 [-0.29 ; 0.04]	-0.13 [-0.30 ; 0.04]	-0.12 [-0.28 ; 0.04]	-0.12 [-0.29 ; 0.04]	-0.14 [-0.30 ; 0.01]	-0.12 [-0.27 ; 0.04]
**City isolation** [Mean distance to the nearest urban patch within the geographic boundary(m)]	0.04 [-0.07 ; 0.16]	0.04 [-0.09 ; 0.17]	0.04 [-0.07 ; 0.16]	0.00 [-0.11 ; 0.10]	0.05 [-0.08 ; 0.17]	0.04 [-0.07 ; 0.16]

The description of the sample for each sensitivity analysis are as follows:
Full model: Model presented in the main text. Excluding Argentina: data sub-set excluding all observations from Argentina as weight and height was self-reported for this country. Excluding population density >3SD: data sub-set excluding observations with population density higher than its 3 standard deviations (SD). Recent surveys: only survey data from 2010 onwards were included in this data sub-set. Excluding BMI outliers: observations with body mass index (BMI) lower that 12 Kg/m2 and higher that 70 Kg/m2 were excluded in this data sub-set. Including interaction terms between Country and individual educational level: as education could modified the association between the built environment characteristics and our health outcomes, we included in this model the interaction terms for this country and individual education level.

Supplementary Table 4b | Sensitivity analysis
Associations between sub-city and city exposures and obesity

Sub-city and city characteristics (z-scores)	Full Model for Obesity	Excluding Argentina	Excluding pop.dens >3SD	Recent surveys only	Excluding BMI outliers	Normal weight vs Overweight + Obese	Including interaction terms between country and individual educational level
	**OR [95% CI]**	**OR [95% CI]**	**OR [95% CI]**	**OR [95% CI]**	**OR [95% CI]**	**OR [95% CI]**	**OR [95% CI]**
**Sub-city intersection density** (n/km2)	1.04 [1.00 ; 1.08]	1.04 [1.00 ; 1.08]	1.03 [0.99 ; 1.07]	1.03 [0.99 ; 1.07]	1.04 [1.00 ;1.08]	1.01 [0.98 ; 1.05]	1.04 [1.00 ; 1.08]
**Sub-city greenness** (median NDVI)	0.97 [0.93 ; 1.01]	0.96 [0.91 ; 1.01]	0.97 [0.93 ; 1.01]	0.97 [0.93 ; 1.02]	0.97 [0.93 ;1.01]	0.98 [0.94 ; 1.03]	0.97 [0.93 ; 1.01]
**Sub-city population density in built up areas** (n/km2)	1.00 [0.95 ; 1.04]	1.01 [0.96 ; 1.06]	1.02 [0.98 ; 1.06]	1.01 [0.97 ; 1.05]	1.00 [0.95 ;1.04]	1.00 [0.96 ; 1.05]	1.010 [0.96 ; 1.04]
**City fragmentation** [Patch density (n/100ha)]♯	0.95 [0.88 ; 1.02]	0.94 [0.87 ; 1.01]	0.95 [0.89 ; 1.02]	0.96 [0.90 ; 1.03]	0.95 [0.88 ;1.02]	1.01 [0.94 ; 1.08]	0.96 [0.89 ; 1.03]
**City isolation** [Mean distance to the nearest urban patch within the geographic boundary(m)]	1.01 [0.96 ; 1.06]	1.01 [0.96 ; 1.06]	1.01 [0.96 ; 1.06]	0.99 [0.95 ; 1.03]	1.01 [0.97 ;1.06]	1.02 [0.96 ; 1.07]	1.01 [0.96 ; 1.06]

Different sensitivity analyses are presented. The description of the sample for each sensitivity analysis are as follows: Full model: Model presented in the main text. Excluding Argentina: data sub-set excluding all observations from Argentina as weight and height was self-reported for this country. Excluding pop.dens >3SD: data sub-set excluding observations with population density higher than its 3 standard deviations (SD). Recent surveys: only survey data from 2010 onwards were included in this data sub-set. Excluding BMI outliers: observations with body mass index (BMI) lower that 12 Kg/m2 and higher that 70 Kg/m2 were excluded in this data sub-set. Normal weight vs Overweight + Obese: this dataset compares subjects with normal weight <25 kg/m2) vs. overweight and obesity (≥25 kg/m2) Including interaction terms between Country and individual educational level: as education could modified the association between the built environment characteristics and our health outcomes, we included in this model the interaction terms for this country and individual education level.

Supplementary Table 4c | Sensitivity analysis
Associations between sub-city and city exposures and Type 2 Diabetes

Sub-city and city characteristics (z-scores)	Full Model for Type 2 Diabetes	Excluding Panama	Excluding pop.dens >3SD	Recent surveys only	Including interaction terms between country and individual educational level
	**OR [95% CI]**	**OR [95% CI]**	**OR [95% CI]**	**OR [95% CI]**	**OR [95% CI]**
**Sub-city intersection density** (n/km2)	1.04 [0.98 ; 1.10]	1.06 [1.01 ; 1.11]	1.04 [0.98 ; 1.10]	1.07 [1.01 ; 1.13]	1.03 [0.97 ; 1.10]
**Sub-city greenness** (median NDVI)	0.98 [0.94 ; 1.02]	0.99 [0.95 ; 1.03]	0.98 [0.94 ; 1.02]	0.99 [0.95 ; 1.04]	0.98 [0.93 ; 1.02]
**Sub-city population density in built up areas** (n/km2)	0.96 [0.92 ; 1.00]	0.96 [0.91 ; 1.01]	.99 [0.93 ; 1.05]	0.99 [0.95 ; 1.04]	0.97 [0.92 ; 1.01]
**City fragmentation** [Patch density (n/100ha)]♯	0.98 [0.92 ; 1.04]	0.98 [0.92 ; 1.05]	0.98 [0.92 ; 1.04]	0.97 [0.92 ; 1.03]	0.99 [0.93 ; 1.06]
**City isolation** [Mean distance to the nearest urban patch within the geographic boundary(m)]	0.99 [0.96 ; 1.03]	0.99 [0.96 ; 1.03]	0.99 [0.96 ; 1.03]	1.00 [0.96 ; 1.04]	0.99 [0.95 ; 1.03]

Different sensitivity analyses are presented. The description of the sample for each sensitivity analysis are as follows:
Full model: Model presented in the main text. Excluding Panama: data sub-set excluding all observations from Panama as the original survey did not distinguish gestational diabetes from Type 2 Diabetes. Excluding pop.dens >3SD: data sub-set excluding observations with population density higher than its 3 standard deviations (SD). Recent surveys: only survey data from 2010 onwards were included in this data sub-set. Including interaction terms between Country and individual educational level: as education could modified the association between the built environment characteristics and our health outcomes, we included in this model the interaction terms for this country and individual education level.
All exposures were scaled based on the mean and standard deviation (SD) and therefore the unit of contrast is the SD. Each model includes all built environment exposures, adjusted for age, sex, education, population educational attainment at sub-city level, percentage of urban area, and country as a fixed effect. BMI: Body mass index: NDVI: Normalized Difference Vegetation Index.

Supplementary Table 5 | General information of the health surveys used for individual level data, i.e., BMI, obesity and type 2 diabetes, as well as, demographic information (age, sex, and highest educational level completed)

Country / Year	Survey	Age Range Sampled	Sampling Strategy	Geographic coverage	Over-sampling	Representation
**Argentina 2013**	Encuesta Nacional de Factores de Riesgo, ENFR (National Risk Factors Survey)	≥18 years	Multistage [Aglomerado censal; área (groups of radio censales); household; person 18 years or older]<br><br>Stratified [population size; education level of head of household]	*Localidades* with over 5,000 population	None	National, four *localidades* groups based on size, 6 regions, 23 provinces, Ciudad Autonoma de Buenos Aires, and 8 metropolitan areas >500,000 population.
**Brasil 2013**	Pesquisa Nacional de Saúde, PNS (National Health Survey)	≥18 years	Multistage [census tracts or groups of census tracts; households; person 18 years or older]<br><br>Stratified [capital city, metropolitan region, or integrated economic development region, then rest of municipalities; Urban/rural; total household income]	Regions (5) States or federation units (27), state capitals (27)	None	Regions (5) States or federation units (27), state capitals (27), urban and rural, metropolitan areas and development integrated areas
**Chile 2010**	Encuesta Nacional de Salud, ENS (National Health Survey)	≥15 years	Multistage [Comunas; Segments within comunas; household; person 15 years or older]<br><br>Stratified [urban/rural with three groups of population sizes]	National	Adults ≥65, regions distinct to Metropolitan Region, rural areas	National, Regions (15), urban/rural
**Colombia 2007**	Encuesta Nacional de Salud, ENS (National Health Survey)	0 – 69 years	Multistage [Municipalities or combination of municipalities if small; Manzanas; household; person adults 18-69 and all children 17 and under]<br><br>Stratified [region; urbanization of municipal seats; urban/rural municipal population; unsatisfied basic needs]	National	None	Region, department, subregion, urban area of municipal capitals, urban/rural, by poverty level

Country / Year	Survey	Age Range Sampled	Sampling Strategy	Geographic coverage	Over-sampling	Representation
**Guatemala 2002**	Encuesta Multinacional de Diabetes Mellitus y Factores de Riesgo, CAMDI (Multinational Survey of Diabetes Mellitus & Risk Factors, Central American Diabetes Initiative)	≥20 years	Multistage [Segmento censal, groups of dwellings (compacto); all household members 20 years and older]	Villa Nueva Municipio, a part of metropolitan Guatemala City	None	Villa Nueva Municipio
**Mexico 2012**	Encuesta Nacional de Salud y Nutricion, ENSANUT (National Survey for Health and Nutrition)	All ages	Multistage [AGEB; Manzana (urban) or pseudo-manzanas withig localidades (rural); Households; 1 person within each of the groups (0-4 years, 5-9 years, 10-19 years, 20 years and older, recent medical service user)]<br>Stratified [socioeconomic status of AGEB at the state level]	National	AGEB with the highest index of poor socioeconomic conditions	National, state, metropolitan areas, urban/rural, high/low SES
**Nicaragua 2003**	Encuesta Multinacional de Diabetes mellitus y Factores de Riesgo, CAMDI (Multinational Survey of Diabetes Mellitus & Risk Factors, Central American Diabetes Initiative)	≥20 years	Multistage [Urban districts divided into 50 strata, groups of households (compacto); all family members living together 20 years and older]	Municipality of Managua	None	Municipality of Managua
**Panama 2007**	Encuesta Nacional de Salud y Calidad de Vida ENSCAVI (National Survey of Health and Quality of Life)	≥18 years	Multistage [Census segments; Dwellings; People ≥18 years]<br><br>Stratified [Indigenous population in province; Urban/rural]	National	None	National, District

Country / Year	Survey	Age Range Sampled	Sampling Strategy	Geographic coverage	Over-sampling	Representation
**Peru 2016**	Encuesta Nacional de Demografia y Salud, ENDES (National Survey of Demographics and Health)	All ages	Multistage [Conglomerado (set of census blocks – urban) or Empadronamiento (set of households – rural); Households; One person within each of the groups (>15 years, females 15-49 years, children <5 years, children <12 years)]<br><br>Stratified [Department; Urban/Rural]	National	None	National, Urban National, Rural National, Natural Region: Lima Metropolitan area, coast/mountain/jungle
**El Salvador 2004**	Encuesta Multinacional de Diabetes mellitus y Factores de Riesgo, CAMDI (Multinational Survey of Diabetes Mellitus & Risk Factors, Central American Diabetes Initiative)	≥20 years	Multistage [Segmento censal, groups of dwellings (compacto); all household members 20 years and older]	Municipio of Santa Tecla	Unknown	Municipio of Santa Tecla

Supplementary Table 6 | Description of exposure and covariates variables used
Variables included in the analysis are listed, as well as their roles, level of measurement, data source, definition and interpretation, and year of data collection.

Role	Level	Variable	Data Source	Definition and interpretation	Year of data collection
**Exposure**	**Sub-city**	**Intersection density (n/km^2)**	Shapefiles for each unit were provided by the SALURBAL data & methods core. The underlying street network was then obtained from OpenStreetMap. Each intersection (a point where more than 2 streets meet) becomes a node of the network and each street segment connecting two intersections becomes an edge in the network.	Measures the amount of intersections per km^2 of area. Sub-city units with higher values have a lot of intersections (and thus streets) by unit of area.	2018
		**Greenness (median NDVI)**	NDVI is from a MODIS vegetation product, MOD13Q1.006 (https://lpdaac.usgs.gov/products/mod13q1v006/), which computed vegetation indices from atmospherically corrected bidirectional surface reflectance that had been masked for water, clouds, heavy aerosols, and cloud shadows. The temporal and spatial resolution of this NDVI product is 16 days and 250 m, respectively. The images are taken twice daily and aggregated every 16 days, by taking the highest NDVI value per image pixel. For the annual value, the 16-days NDVI value, was combined into a single image, by taking the maximum value per image pixel from all available 16-day observations. The NDVI data has a global coverage from year 2000 to the present.<br>For the purpose of the analyses, we used the zonal median of annual maximum NDVI in urbanized areas for the district unit (sub-city level), as a summary measure, where, for each image pixel, we calculate the maximum from all observations in a year. Then, we took the area-level median of these maximums as the NDVI for a given geography. Seasonal greenness is	Zonal median of annual maximum NDVI in urbanized area (excluding water and vegetation). NDVI is an indicator of vegetation 'greenness'. NDVI ranges from -1 to 1, with higher values indicating more vegetated areas that are also greener.	We selected the data to match the year of the survey where the outcome was assessed (ie: if survey is in 2010, then, we use NDVI from 2010).

			accounted for by selecting the highest NDVI value of the year. Additionally, we selected the data to match the year of the survey where the outcome was assessed (i.e.: if survey is in 2010, then, we use NDVI from 2010).		
		**Population density (n/km^2)**	Calculated as population divided by build-up area. Population data was calculated as the sum of Global Urban Footprint (GUF) and Facebook's Population Density Maps both in 30m x 30m grid cells). Built-up area for sub-city units were calculated by adding the area of all 30m grid cells within the sub-city unit from both products.	Population per km2 in all the urban patches inside the geographic boundary. Population is adjusted for United Nations' country-level population projections. A higher value indicates a denser urban development pattern.	2010
	**City**	**Fragmentation [Patch density (n/100ha)]**	The urban footprint data (in 30m x 30m grid cells) comes from the "hole-filled" Global Urban Footprint (GUF). This variable is calculated based on 30m x 30m grid cells using the FRAGSTATS 4.2 software package, as follows: total number of urban patches of the city divided by the area of 30m x 30m grid cell within the city.	Number of urban patches divided by the total area of the geographic unit (in 100 hectares). Higher patch density reflects fragmentation of urban expansion, when the variable percentage of urban area is included as covariate.	2012
		**Isolation [Area-weighted Mean Nearest Neighbor Distance (m)]**	The urban footprint data (in 30m x 30m grid cells) comes from the "hole-filled" Global Urban Footprint (GUF). This variable is calculated based on 30m x 30m grid cells using the FRAGSTATS 4.2 software package, as follows: the sum of the nearest neighbor distance of urban parch in a city divided by the number of urban patches of the city.	Mean distance (in meters) to the nearest urban patch within the geographic boundary. This value is weighted by the area of each patch. Indicator of how spread out are urban settlements within the geographic. A higher value indicates higher level of isolation.	2012
**Covariates**	**Individual**	**Age**	Health survey	Self-reported age assessed by respondent age in years or calculated using respondent birthday. For Multi-level regression/logistic models, age in units of 10.	Argentina (2013) Brazil (2013) Chile (2010) Colombia (2007) Guatemala
		**Sex**	Health survey	A survey participant is defined as male if they have a response of "varón,"	

			"masculino," or "hombre." A survey participant is defined as female if they have a response of "mujer" or "femenino."	(2002) Mexico (2012) Nicaragua (2003) Panama (2007) Peru (2016) El Salvador (2004)
	**Highest educational level completed**	Health survey	Four categories are defined: less than primary, primary, secondary and university or higher.	
**Sub-city**	**Population educational attainment (Z-score)**	Health survey	A summary score of sub-city education (summed Z score for the percentage of population age 25 or above that has completed high school level or above, and the percentage of population age 25 or above that completed university level or above) as a marker of socioeconomic context.	
**City**	**Percentage of urban area (%)**	The urban footprint data (in 30m x 30m grid cells) comes from the "hole-filled" Global Urban Footprint (GUF). This variable is calculated based on 30m x 30m grid cells using the FRAGSTATS 4.2 software package, as follows: total urban area (built up area) divided by the total area of the city, and multiplied by 100.	Ranges from 0 to 100. It is used as an adjustment variable for fragmentation in order to account for some surrounding non-built up areas sub-city units, which could introduce measurement error due to the heterogeneity in urbanized areas. Large value indicates a higher level of built-up areas within the geographic unit.	2012

NDVI: Normalized Difference Vegetation Index

Supplementary Table 7 | Correlation matrix among variables of interest
Correlations between exposure variables using Pearson. The highest correlation was seen between city percentage of urban area with city fragmentation (r= 0.7); however, the former variable is used for adjustment in order to account for some surrounding non-built up areas sub-city units used in the fragmentation calculation, which could introduce measurement errors due to the heterogeneity in urbanized areas

	Sub-city Intersection density	Sub-city Greenness	Sub-city Population Density in built up areas	City Fragmentation	City Isolation	Sub-city Population educational attainment	City Percentage of urban area
**Sub-city Greenness**	-0.4563						
**Sub-city Population Density in built up areas**	0.4897	-0.1288					
**City Fragmentation**	0.4134	0.0990	0.2765				
**City Isolation**	-0.3396	0.0260	-0.2107	-0.4354			
**Sub-city Population educational attainment**	0.3552	-0.2217	0.1512	0.0780	-0.2092		
**City Percentage urban area**	0.6164	-0.1941	0.4777	0.7068	-0.4217	0.1362	
**Age**	0.0415	-0.0373	-0.0365	0.0527	-0.0127	-0.0041	0.0482
