# Supplementary material for: The urban built environment and adult BMI, obesity, and diabetes in Latin American cities
Source: Nat Commun. 2022 Dec 29;13:7977. doi: 10.1038/s41467-022-35648-w (PMC9800402; doi:10.1038/s41467-022-35648-w)
Supplement: Supplementary file 3 — Reporting Summary [file 41467_2022_35648_MOESM3_ESM.pdf]

nature portfolio

Corresponding author(s): Cecilia Anza-Ramirez and J. Jaime Miranda

Last updated by author(s): Dec 6, 2022

Reporting Summary

Nature Portfolio wishes to improve the reproducibility of the work that we publish. This form provides structure for consistency and transparency in reporting. For further information on Nature Portfolio policies, see our Editorial Policies and the Editorial Policy Checklist.

Statistics

For all statistical analyses, confirm that the following items are present in the figure legend, table legend, main text, or Methods section.

n/a	Confirmed	
☐	☒	The exact sample size (*n*) for each experimental group/condition, given as a discrete number and unit of measurement
☐	☒	A statement on whether measurements were taken from distinct samples or whether the same sample was measured repeatedly
☐	☒	The statistical test(s) used AND whether they are one- or two-sided<br>*Only common tests should be described solely by name; describe more complex techniques in the Methods section.*
☐	☒	A description of all covariates tested
☐	☒	A description of any assumptions or corrections, such as tests of normality and adjustment for multiple comparisons
☐	☒	A full description of the statistical parameters including central tendency (e.g. means) or other basic estimates (e.g. regression coefficient) AND variation (e.g. standard deviation) or associated estimates of uncertainty (e.g. confidence intervals)
☐	☒	For null hypothesis testing, the test statistic (e.g. *F*, *t*, *r*) with confidence intervals, effect sizes, degrees of freedom and *P* value noted<br>*Give P values as exact values whenever suitable.*
☒	☐	For Bayesian analysis, information on the choice of priors and Markov chain Monte Carlo settings
☐	☒	For hierarchical and complex designs, identification of the appropriate level for tests and full reporting of outcomes
☒	☐	Estimates of effect sizes (e.g. Cohen's *d*, Pearson's *r*), indicating how they were calculated

Our web collection on statistics for biologists contains articles on many of the points above.

Software and code

Policy information about availability of computer code

Data collection	No software was used
Data analysis	All analyses were conducted in SAS/STAT® software v9.4 (SAS Institute Inc., Cary, NC, USA). The code is available on GitHub at https://github.com/Drexel-UHC/SALURBAL-MS62.

For manuscripts utilizing custom algorithms or software that are central to the research but not yet described in published literature, software must be made available to editors and reviewers. We strongly encourage code deposition in a community repository (e.g. GitHub). See the Nature Portfolio guidelines for submitting code & software for further information.

Data

Policy information about availability of data

All manuscripts must include a data availability statement. This statement should provide the following information, where applicable:

- Accession codes, unique identifiers, or web links for publicly available datasets
- A description of any restrictions on data availability
- For clinical datasets or third party data, please ensure that the statement adheres to our policy

The SALURBAL study obtained health survey data from health and/or statistical agencies within each country. Data from Brazil (https://www.ibge.gov.br/estatisticas/sociais/saude/9160-pesquisa-nacional-de-saude.html), Chile (http://epi.minsal.cl/encuesta-ens/), Mexico (https://ensanut.insp.mx/), Panama (http://www.gorgas.gob.pa/enscavi/), Peru (http://webinei.inei.gob.pe/anda_inei/index.php/catalog/563), and El Salvador (http://www.datos.gob.sv/dataset/encuesta-nacional-de-enfermedades-cronicas) are publicly available at the links provided. Data from Argentina, Colombia, Guatemala, and Nicaragua are available under

restricted access due to data use agreements between the SALURBAL Study and statistical agencies within the country. Requests for the harmonized dataset can be obtained by contacting the SALURBAL project salurbal.data@drexel.edu and after completing a data use agreements. Requests are reviewed by the Data Methods Core and Publications & Presentations Committee on a monthly basis. To learn more about SALURBAL's dataset, visit https://drexel.edu/lac/ or contact the project at salurbal@drexel.edu. Analytic code can be found at https://github.com/Drexel-UHC/SALURBAL-MS62.

Human research participants

Policy information about studies involving human research participants and Sex and Gender in Research.

Reporting on sex and gender	Sex, a self-reported variable, was considered in the study design as a covariate. Please, refer to Supplementary Table 6 for further information on the variable. Descriptive analyses are shown by sex, while our association of interest are adjusted by this variable.
Population characteristics	Table 1 shows participant characteristics according to obesity and T2D status. Overall, the average age was 42 years old, around 58% of the sample was female, and half of the sample completed high school education or more. Respondents with obesity, as well as with T2D, tended to be older and have lower educational levels when compared with respondents without obesity or T2D, respectively (Table 1). Our characterization of the study sample by country and by population density is available in Supplementary Tables 2 and 3, respectively.
Recruitment	N/A
Ethics oversight	The SALURBAL study protocol has been approved by the Drexel University Institutional Review Board with ID #1612005035.

Note that full information on the approval of the study protocol must also be provided in the manuscript.

Field-specific reporting

Please select the one below that is the best fit for your research. If you are not sure, read the appropriate sections before making your selection.

☒ Life sciences ☐ Behavioural & social sciences ☐ Ecological, evolutionary & environmental sciences

For a reference copy of the document with all sections, see nature.com/documents/nr-reporting-summary-flat.pdf

Life sciences study design

All studies must disclose on these points even when the disclosure is negative.

Sample size	The SALURBAL project (Salud Urbana en America Latina/Urban Health in Latin America) compiled and harmonized social, built environment and health survey data to examine multilevel aspects of health across all cities with ≥ 100,000 inhabitants as of 2010 in 11 Latin American countries (Argentina, Brazil, Chile, Colombia, Costa Rica, Guatemala, Mexico, Nicaragua, Panama, Peru, and El Salvador). A city was defined as the combination of administrative units (i.e. sub-city units) that overlap with the urban extent of the city, as determined from satellite imagery. Thus, a city may include a single administrative unit or a combination of adjacent administrative units.67 Using this approach, 371 cities were identified and operationalized as clusters of the smallest administrative units (1436 sub-city units, most commonly named municipios, comunas or distritos). The SALURBAL data resource includes several harmonized health survey data (non-communicable disease risk factors, adult and children) and geolinked to vast built, natural, and social environment data for at up to three levels: cities, sub-city units (e.g. Municipios of counties that compose cities), and neighbourhoods (similar to US census tracts). In this cross-sectional multilevel analysis, we focused on adults 18 years old and older, living in sub-city units and cities for which we were able to identify and link selected social and built environment data with individual-level T2D, obesity, and demographic information. The analytical data set comprised data from the following nationally representative health surveys, with years given in brackets: Argentina (2013), Brazil (2013), Chile (2010), Colombia (2007), Guatemala (2002), Mexico (2012), Nicaragua (2003), Panama (2007), Peru (2016), and El Salvador (2004) (Figure 1).
Data exclusions	For all countries, T2D was defined as self-reported physician diagnosis of high blood sugar levels or diabetes. Although gestational diabetes confers an increased lifetime risk of T2D, the questionnaires did not clearly distinguish between gestational diabetes and later progression to T2D. As such, given that our research question was focused on the built environment as it relates to T2D, we excluded women who answered yes to a gestational diabetes diagnosis. This exclusion criterium was pre-established before data analysis.
Replication	The statistical analysis code is available on GitHub at https://github.com/Drexel-UHC/SALURBAL-MS62 for replication.
Randomization	This study analysed harmonised data available from different countries at different levels, i.e. individual level from health surveys and sub-city and city level, from different sources. No experiments were conducted in this study.
Blinding	There was no group allocation involved in this study.

Reporting for specific materials, systems and methods

We require information from authors about some types of materials, experimental systems and methods used in many studies. Here, indicate whether each material, system or method listed is relevant to your study. If you are not sure if a list item applies to your research, read the appropriate section before selecting a response.

Materials & experimental systems

n/a	Involved in the study
☒	☐ Antibodies
☒	☐ Eukaryotic cell lines
☒	☐ Palaeontology and archaeology
☒	☐ Animals and other organisms
☒	☐ Clinical data
☒	☐ Dual use research of concern

Methods

n/a	Involved in the study
☒	☐ ChIP-seq
☒	☐ Flow cytometry
☒	☐ MRI-based neuroimaging
